# Impact of corticosteroids and immunosuppressive therapies on symptomatic SARS-CoV-2 infection in a large cohort of patients with chronic inflammatory arthritis

**DOI:** 10.1186/s13075-020-02395-6

**Published:** 2020-12-30

**Authors:** Ennio Giulio Favalli, Serena Bugatti, Catherine Klersy, Martina Biggioggero, Silvia Rossi, Orazio De Lucia, Francesca Bobbio-Pallavicini, Antonella Murgo, Silvia Balduzzi, Roberto Caporali, Carlomaurizio Montecucco

**Affiliations:** 1 Division of Clinical Rheumatology, ASST Gaetano Pini-CTO Institute, Via Gaetano Pini, 9, 20122 Milan, Italy; 2 Division of Rheumatology, IRCCS Policlinico San Matteo Foundation, Pavia, Italy; 3 Department of Internal Medicine and Therapeutics, University of Pavia, Pavia, Italy; 4 Unit of Clinical Epidemiology & Biometry, IRCCS Policlinico San Matteo Foundation, Pavia, Italy; 5 Department of Clinical Sciences & Community Health, Research Center for Adult and Pediatric Rheumatic Diseases, Università degli Studi di Milano, Milan, Italy

**Keywords:** COVID-19, SARS-CoV-2, Rheumatic diseases, Biologic drugs, Glucocorticoids

## Abstract

**Background:**

Prevalence and outcomes of coronavirus disease (COVID)-19 in relation to immunomodulatory medications are still unknown. The aim of the study is to investigate the impact of glucocorticoids and immunosuppressive agents on COVID-19 in a large cohort of patients with chronic immune-mediated inflammatory arthritis.

**Methods:**

The study was conducted in the arthritis outpatient clinic at two large academic hospitals in the COVID-19 most endemic area of Northern Italy (Lombardy). We circulated a cross-sectional survey exploring the prevalence of severe acute respiratory syndrome-coronavirus-2 nasopharyngeal swab positivity and the occurrence of acute respiratory illness (fever and/or cough and/or dyspnea), administered face-to-face or by phone to consecutive patients from 25 February to 20 April 2020. COVID-19 cases were defined as confirmed or highly suspicious according to the World Health Organization criteria. The impact of medications on COVID-19 development was evaluated.

**Results:**

The study population included 2050 adults with chronic inflammatory arthritis receiving glucocorticoids, conventional-synthetic (cs), or targeted-synthetic/biological (ts/b) disease-modifying drugs (DMARDs). Laboratory-confirmed COVID-19 and highly suspicious infection were recorded in 1.1% and 1.4% of the population, respectively. Treatment with glucocorticoids was independently associated with increased risk of COVID-19 (adjusted OR [95% CI] ranging from 1.23 [1.04–1.44] to 3.20 [1.97–5.18] depending on the definition used). Conversely, patients treated with ts/bDMARDs were at reduced risk (adjusted OR ranging from 0.46 [0.18–1.21] to 0.47 [0.46–0.48]). No independent effects of csDMARDs, age, sex, and comorbidities were observed.

**Conclusions:**

During the COVID-19 outbreak, treatment with immunomodulatory medications appears safe. Conversely, glucocorticoids, even at low-dose, may confer increased risk of infection.

**Trial registration:**

Retrospectively registered. Not applicable.

## Background

The 2019 coronavirus disease (COVID-19) caused by the novel beta-coronavirus severe acute respiratory syndrome coronavirus (SARS-CoV)-2 has a highly variable course ranging from asymptomatic or paucisymptomatic subsets to severe interstitial pneumonia rapidly evolving into acute respiratory distress syndrome (ARDS) [1, 2], which has so far already resulted in the deaths of more than one million people worldwide. The identification of risk factors of COVID-19 critical disease remains imperative in order to better allocate medical resources and develop data-driven guidelines for more vulnerable patients. At present, worse prognosis appears mostly related to increasing age, obesity, and presence of cardiovascular comorbidities [3, 4]. The outcomes of SARS-CoV-2 infection in unique patient populations, such as immunocompromised adults, are in contrast only partly understood [5–9].

COVID-19 and immunosuppression are indeed coupled by a complex and possibly bidirectional relationship [10–13]. On the one hand, in fact, glucocorticoids (GC), cytokine blockers, or small molecules inhibiting Janus kinases (JAK) could theoretically mitigate the hyper-immune reactions responsible of more severe manifestations of COVID-19 [10, 11]. Accordingly, immunomodulating agents are currently being trialed in the treatment of SARS-CoV-2 infection [12–15]. However, concern remains on the possible impact of these same immunosuppressive therapies on increasing the risk of COVID-19 or worsening its clinical course in patients on chronic treatment for immune-mediated inflammatory diseases (IMIDs) [16–19]. Due to a general impairment of the immune system, IMIDs per se are characterized by an intrinsic increased infectious risk [20–22]. In addition, the iatrogenic effect generated by the use of immunomodulating drugs should also be considered [23, 24]. As IMIDs are highly prevalent in Western societies (approximately 5–7%) [25] and routine use of GC and synthetic and biological disease-modifying drugs has become widespread in rheumatology, gastroenterology, and dermatology [26–31], understanding the real impact of immunosuppression on COVID-19 diffusion and severity undoubtedly represents a crucial issue of inter-disciplinary relevance.

In the absence of analyses from large claims databases, guidance on the management of immunosuppressive therapies during the COVID-19 pandemic remains mostly supported by low-quality evidence [32–34]. Preliminary observational studies appear overall reassuring on the incidence and clinical course of COVID-19 in immunosuppressed patients [32–37]. However, different classes of immunomodulating agents, often used in combination in patients with IMIDs, may have a different and even opposite impact on SARS-CoV-2 infection [38–41], which is at present undetermined. Furthermore, previous comparisons of small patient cohorts with the general population hamper definitive conclusions on the specific effects, if any, of disease- and treatment-related risk factors. In order to assist a more informed management of immunosuppressive therapies during the pandemic, here we evaluated the frequency and the characteristics of symptomatic SARS-CoV-2 infection in relation with the use of different immunosuppressing agents on the background of a common group of IMID as rheumatic and musculoskeletal diseases (RMDs).

## Methods

### Study population

The study population included all adult (> 18 years-old) patients diagnosed with rheumatoid arthritis (RA), undifferentiated arthritis (UA), psoriatic arthritis (PsA), or spondyloarthritis (SpA) as diagnosed by the treating rheumatologist, with a follow-up visit scheduled in the period between 25 February and 20 April 2020 at the outpatient clinic of the Division of Clinical Rheumatology of ASST Gaetano Pini-CTO Institute in Milan or of the Rheumatology Department of Policlinico San Matteo in Pavia. The current analysis was approved by the Ethics Committees of the Gaetano Pini Institute and Policlinico San Matteo as part of a project to collect observational data from rheumatological patients followed at the two involved rheumatology units. All included patients have signed an informed consent to participate in the data collection and to data publication. The possibility of including this survey within the abovementioned data collection project has been waived by the same ethics committee.

### Outcomes

A cross-sectional survey was designed to investigate the prevalence of COVID-19 on the study population. The survey comprised two separate sections, one filled in by the rheumatologist and one by the patient. In the first part of the questionnaire, the diagnosis and demographics, the ongoing treatment (both rheumatological and non-rheumatological), the degree of disease activity (measured by Disease Activity Score 28 [DAS28] for RA and UA, Disease Activity in Psoriatic Arthritis [DAPSA] score for PsA, and Ankylosing Spondylitis Disease Activity Score [ASDAS] for spondyloarthritis, where applicable), and the presence of comorbidities were evaluated. The second section investigated confirmed diagnosis of COVID-19 formulated by nasopharyngeal swab, the patient’s contacts with subjects diagnosed with COVID-19, the reported symptoms suggesting viral infection in patients who did not have access to swab (at least one between fever > 37.5 °C, cough, or dyspnea of recent onset), and the patient’s behavior regarding any precautions taken to prevent the contagion. In particular, questions were asked about the use of masks and gloves, social distancing, changes in work activity with the introduction of home working, and adherence to the authorities’ instructions to prevent the contagion. The survey was administered to all patients followed up at the outpatient clinic of the two involved centers, either face-to-face during each visit or by telephone to all patients who missed a scheduled visit during the reporting period. All the information in the second section has been referred to the period between 14 days (the length of the incubation period established by the Italian health authorities) before the start of the survey and the end of the data collection.

According to the WHO definitions (https://www.who.int/docs/default-source/coronaviruse/situation-reports/20200321-sitrep-61-covid-19.pdf?sfvrsn=ce5ca11c_2), we defined a person with laboratory confirmation by nasopharyngeal swab of virus causing COVID-19 infection, irrespective of clinical signs and symptoms, as *confirmed COVID-19*; fever > 37.5 °C and/or cough and/or dyspnea of recent onset in a patient having been in close contact with a confirmed COVID-19 case in the last 14 days prior to onset of symptoms as *highly suspicious COVID-19*; and fever > 37.5 °C and/or cough and/or dyspnea of recent onset in a patient not having been in close contact with a confirmed COVID-19 case in the last 14 days prior to onset of symptoms as *unlikely COVID-19*.

### Statistical analysis

The Stata software was used for computation (Release 16.1, StataCorp, College Station, TX, USA). A 2-sided test was considered statistically significant. Continuous data were described with the mean and standard deviation (SD) or the median and the interquartile range (IQR) depending on the distribution and categorical variables with counts and percent. To maintain data integrity, missing data were not imputed. Percents of missing data were < 5% for all variables except for body mass index, smoking status, and disease activity (< 10%). Outcomes of interest included the following: (i) confirmed COVID-19 only; (ii) confirmed or highly suspicious COVID-19 grouped together; and (iii) confirmed, highly suspicious, or unlikely COVID-19 grouped together. For all the outcomes analyzed, the comparator group included all the remaining cases. Since a number of respiratory pathogens other than SARS-CoV-2 were circulating in Italy at the time of the pandemic, unlikely COVID-19 was also analyzed as a separate outcome after exclusion of confirmed and highly suspicious COVID-19. In this case, the comparator group included non-symptomatic patients. The association of a series of risk factors and of treatment with the outcomes was assessed with logistic regression models. The independent role of treatments was further assessed in a multivariable including home lockdown (the main preventive measure) as well as non-collinear variables with *p* < 0.1 at the univariable analysis. Accuracy of the regression models was tested with the area under the receiver operating characteristic curve (AUC). To avoid overfitting, the number of variables included in multivariable models followed the 1:10 rule with the observed outcome (1 predictive variable for every 10 events). Accordingly, for the outcome of confirmed COVID-19 (*n* = 23 cases), significant variables at univariable regression could be only tested in multiple bivariable models.

### Role of the funding source

No specific funding was received from any funding bodies in the public, commercial, or not-for-profit sectors to carry out the work described in this manuscript.

## Results

### Study population

**Fig. 1:**
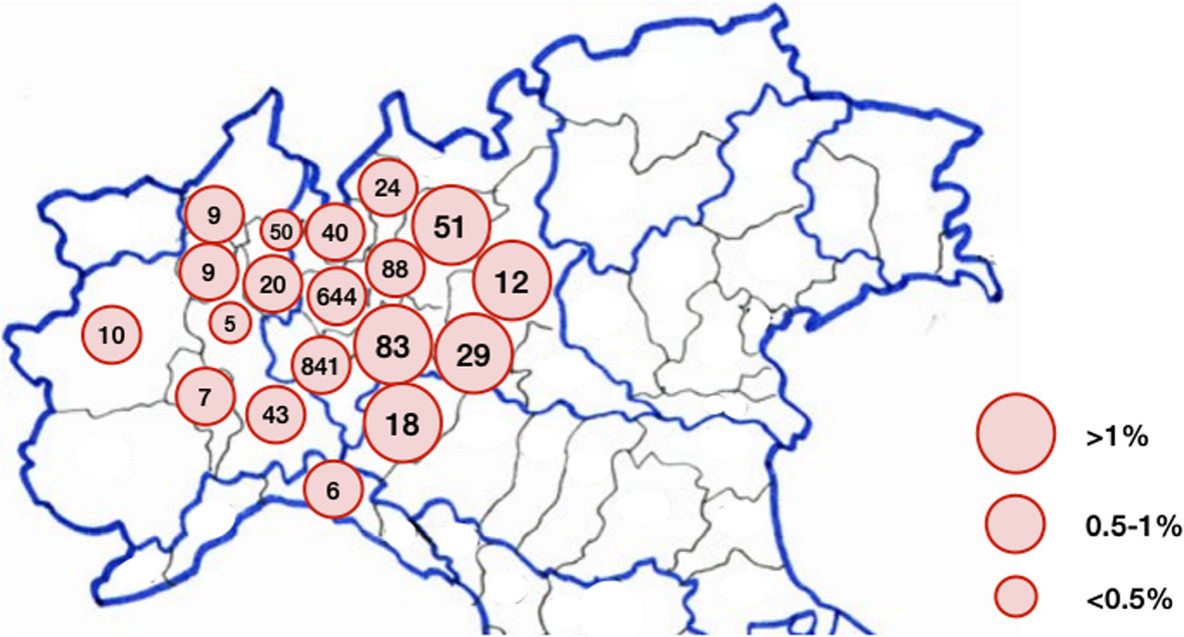
Patient disposition according to the province of residence. The size of the balloon indicates the incidence of COVID-19 in the province. The number in the balloon expresses the number of patients enrolled in each province (in the figure only the provinces with at least 5 patients enrolled were considered)

During the evaluation period, 2091 patients were surveyed. The rate of non-responders was 1.96% and, among them, the great majority (35 out of 41, 85.3%) were confirmed to be alive and without signs of infections by a relative, although they could not be reached directly for a telephone interview. These patients were not included in the study population, which finally encompassed 2050 subjects. Demographic and clinical characteristics of the study population are summarized in Table 1. Mean (± SD) age was 58 (± 15) years, and the majority of the patients were female (66%). Age and gender distribution within different diagnostic subgroups were as expected according to the specific type of arthritis. Nearly all (95.4%) had residency in a Province of Northern Italy with COVID-19 incidence ≥ 0.5%, with approximately 10% residing in high-risk areas (incidence ≥ 1%) (Fig. 1). Patients had established arthritis of long duration (median 10 years), and 62.3% were on treatment with biologic (b) or targeted synthetic (ts) disease-modifying anti-rheumatic drugs (DMARDs) (a tumor necrosis factor (TNF) antagonist in the majority of the cases), alone or in combination with conventional synthetic (cs) DMARDs. A small proportion of our cohort (18.6%) was receiving hydroxychloroquine (HCQ). Detailed treatment disposition is shown in supplementary Table S1. Approximately one third of the patients was on concurrent chronic treatment with GC. No significant difference in demographic, clinical, and treatment characteristics was observed between the two recruiting centers (supplementary Table S2).

**Table 1:** Characteristics of the study population

	Total ***n*** = 2.050	RA ***n*** = 1.228	UA ***n*** = 127	PsA ***n*** = 398	SpA ***n*** = 297
Age, mean (SD), years	57.8 (14.9)	61.4 (14.5)	57.8 (17.1)	54.6 (12.6)	47.5 (12.7)
Female gender, *n* (%)	1.354 (66)	943 (76.8)	91 (71.7)	196 (49.2)	124 (41.8)
Current smokers, *n* (%)	276 (16.5)	147 (14.6)	23 (19.3)	57 (18.3)	49 (20.7)
BMI, mean (SD)	25.2 (5.1)	25.0 (5.1)	25.7 (4.8)	26 (5.9)	24.8 (4.1)
Overweight, *n* (%)	525 (32.5)	304 (31)	39 (31.5)	102 (35.3)	80 (36)
Obese, *n* (%)	233 (14.4)	130 (13.3)	25 (20.2)	58 (20.1)	20 (9)
Hypertension, *n* (%)	643 (32.5)	430 (36.2)	45 (35.7)	113 (29.8)	55 (19.2)
Diabetes, *n* (%)	158 (8)	109 (9.2)	6 (4.8)	31 (8.2)	12 (4.2)
Disease duration, median (IQR), years	10 (5–16)	10 (5.9–18)	3.8 (2–6)	10 (5–15)	10 (5–16)
Use of PDN, *n* (%)	641 (31.3)	511 (41.6)	29 (22.8)	70 (17.6)	31 (10.4)
PDN dose, mean (SD), mg/day	4.3 (3.3)	4.3 (3.1)	3.3 (2.2)	4.9 (4.2)	4.7 (4.5)
< 2.5 mg/day, *n* (%)	57 (8.9)	45 (8.8)	5 (17.2)	5 (7.1)	2 (6.5)
2.5–5 mg/day, *n* (%)	522 (81.4)	417 (81.6)	23 (79.4)	55 (78.6)	27 (87)
> 5 mg/day, *n* (%)	62 (9.7)	49 (9.6)	1 (3.4)	10 (14.3)	2 (6.5)
Use of HCQ, *n* (%)	382 (18.6)	284 (23.1)	72 (56.7)	21 (5.3)	5 (1.7)
Use of csDMARDs, *n* (%)	1.048 (51.2)	733 (59.7)	24 (19)	217 (54.5)	74 (24.9)
MTX, *n* (%)	889 (43.4)	649 (52.9)	17 (13.5)	175 (44)	48 (16.2)
SSZ, *n* (%)	98 (4.8)	32 (2.6)	7 (5.6)	35 (8.8)	24 (8.1)
LFN, *n* (%)	53 (2.6)	45 (3.7)	0 (0)	6 (1.5)	2 (0.7)
Others, *n* (%)	27 (1.3)	16 (1.3)	0 (0)	9 (2.3)	2 (0.7]
Use of b/tsDMARDs, *n* (%)	1.278 (62.3)	735 (59.9)	0 (0)	291 (73.1)	252 (84.8)
TNF antagonists	743 (36.2)	339 (27.6)		186 (46.7)	218 (73.4)
IL6-R antagonists	136 (6.6)	136 (11.1)		0 (0)	0 (0)
IL17-IL23 antagonists	95 (4.6)	1 (0.08)		63 (15.8)	31 (10.4)
CTLA4 Ig	146 (7.1)	145 (11.8)		1 (0.3)	0 (0)
Rituximab	14 (0.7)	14 (1.1)		0 (0)	0 (0)
JAK inhibitors	93 (4.5)	91 (7.4)		1 (0.3)	1 (0.3)
IL1-R antagonists	8 (0.4)	8 (0.7)		0 (0)	0 (0)
Others	43 (2.1)	1 (0.08)		40 (10.1)	2 (0.7)

*RA* rheumatoid arthritis, *UA* undifferentiated arthritis, *PsA* psoriatic arthritis, *SpA* spondyloarthritis, *BMI* body mass index, *PDN* prednisone, *HCQ* hydroxychloroquine, *csDMARDs* conventional synthetic disease-modifying anti-rheumatic drugs, *MTX* methotrexate, *SSZ* sulfasalazine, *LFN* leflunomide, *b/tsDMARDs* biological/targeted synthetic disease-modifying anti-rheumatic drugs, *TNF* tumor necrosis factor, *IL* interleukin, *R* receptor, *CTLA4* cytotoxic T lymphocyte antigen 4, *JAK* Janus kinase

### Frequency of SARS-CoV-2 infection

During the observation period, 23 patients (1.1%) were diagnosed with COVID-19 according to nasopharyngeal-swab (confirmed COVID-19). Twenty-nine additional patients (1.4%) reported acute respiratory illness having been in close contact with a confirmed COVID-19 case in the last 14 days prior to onset of symptoms (highly suspicious COVID-19). Two-hundred and sixty-one (12.7%) instead described respiratory symptoms without known contact with a positive COVID-19 case (unlikely COVID-19).

Table 2 summarizes the demographic and clinical characteristics of SARS-CoV-2 infection according to the different definitions. Of the 10 patients developing COVID-19 in course of b/tsDMARD treatment, 6 were receiving a TNF antagonist, 2 a JAK inhibitor, 1 abatacept, and 1 secukinumab. The b/tsDMARD was taken in combination with a csDMARD in 5 cases (3 methotrexate (MTX), 1 sulfasalazine (SSZ), and 1 leflunomide (LFN)). Four patients were on concurrent HCQ treatment. Of the cases developing COVID-19 in the absence of b/tsDMARD therapy, 8 were on MTX, 1 on SSZ, 1 on cyclosporin, and 1 on HCQ; none was receiving more than 1 csDMARD in combination. The two patients receiving neither a csDMARD nor HCQ were taking GC. Eleven patients (47.8%) required hospitalization with low-flow oxygen supplementation, and none was managed in intensive care unit; all recovered and were readmitted home. Of the 29 cases of highly probable SARS-CoV-2 infections, 11 (37.9%) were on b/tsDMARD treatment (45.4% on a TNF antagonist, 27.3% on a JAK inhibitor, 9.1% on secukinumab, 9.1% on tocilizumab, 9.1% on others), which was taken in combination with a csDMARD in 27.3% of the cases (MTX in all). One patient was receiving HCQ. Of the 18 highly suspicious cases not being treated with b/tsDMARDs, 6 were on MTX, 2 on MTX + HCQ, 2 on SSZ, and 2 on HCQ. Finally, treatment disposition of the 261 patients with acute respiratory symptoms without known contacts with COVID-19 cases was similar to that of non-COVID-19 cases.

**Table 2:** Frequency and patients’ disposition of SARS-CoV-2 infection

	Confirmed COVID-19	Highly suspicious COVID-19	Unlikely COVID-19	Non-COVID-19
	***n*** = 23	***n*** = 29	***n*** = 261	***n*** = 1737
Age, mean (SD), years	62.8 (13.1)*^§^	52.8 (13.1)*^§^	53.8 (14.6)*^§^	58.5 (14.9)*^§^
Female gender, *n* (%)	15 (65.2)	21 (72.4)	178 (68.2)	1140 (65.6)
Current smokers, *n* (%)	4 (21.1)	3 (11.5)	38 (18.3)	231 (16.2)
BMI, median (IQR)	26.3 (7)	24.6 (4.3)	24.9 (5.4)	25.3 (5.1)
Overweight, *n* (%)	4 (28.6)	7 (25.9)	53 (26.1)^#^	461 (33.6)^#^
Obese, *n* (%)	3 (21.4)	4 (14.8)	33 (16.3)	193 (14.1)
Hypertension, *n* (%)	10 (50)	10 (37)	76 (30.6)	547 (32.5)
Diabetes, *n* (%)	2 (10)	1 (3.7)	22 (8.9)	133 (7.9)
Diagnosis				
SpA, *n* (%)	4 (17.4)	5 (17.2)	48 (18.4)	240 (13.8)
PsA, *n* (%)	2 (8.7)	4 (13.8)	52 (19.9)	340 (19.6)
UA, *n* (%)	0 (0)	4 (13.8)	11 (4.2)	112 (6.4)
RA, *n* (%)	17 (73.9)	16 (55.2)	150 (57.5)	1045 (60.2)
Disease duration, median (IQR), months	96 (60–153)	96 (50–147)	120 (60–180)	120 (60–192)
≤ 5 years, *n* (%)	7 (30.4)	10 (35.4)	70 (27.1)	496 (28.7)
5–10 years, *n* (%)	8 (34.8)	12 (41.4)	66 (25.6)	449 (26)
10–15 years, *n* (%)	6 (26.1)	0 (0)	63 (24.4)	361 (20.9)
> 15 years, *n* (%)	2 (8.7)	7 (24.1)	59 (22.9)	422 (24.4)
Use of PDN, *n* (%)	13 (56.5)**	9 (31)	73 (28)**	549 (31.7)**
PDN dose, mean (SD), mg/day	4.6 (1.4)	3.9 (2.2)	4.3 (2.1)	4.3 (3.5)
< 2.5 mg/day, *n* (%)	0 (0)	2 (22.2)	6 (8.2)	49 (9)
≥ 2.5 mg/day, *n* (%)	13 (100)	7 (77.8)	67 (91.8)	498 (91)
Use of HCQ, *n* (%)	5 (21.7)	5 (17.2)	47 (18)	325 (18.7)
Use of csDMARDs, *n* (%)	15 (65.2)	13 (44.8)	153 (54.8)	877 (50.5)
Use of b/tsDMARDs, *n* (%)	10 (43.5)**	11 (37.9)^^^	183 (70.1)**^^^	1074 (61.8)^^^

*BMI* body mass index, *RA* rheumatoid arthritis, *UA* undifferentiated arthritis, *PsA* psoriatic arthritis, *SpA* spondyloarthritis, *PDN* prednisone, *HCQ* hydroxychloroquine, *csDMARDs* conventional synthetic disease-modifying anti-rheumatic drugs, *b/tsDMARDs* biological/targeted synthetic disease-modifying anti-rheumatic drugs

*p < 0.05 for confirmed COVID-19 compared to all the other groups

^§^*p* < 0.05 for non-COVID-19 compared to all the other groups

^#^*p* < 0.05 for non-COVID-19 compared to unlikely COVID-19

**p < 0.05 for confirmed COVID-19 compared to unlikely COVID-19

^^^*p* < 0.05 for probable COVID-19 compared to unlikely COVID-19 and non-COVID-19

### Factors associated with SARS-CoV-2 infection

#### Confirmed infection

Results from univariable analysis are presented in Table 3. None of the demographic characteristics was significantly associated with laboratory-confirmed SARS-CoV-2 infection in our patients. The presence of hypertension conferred an OR (95% CI) of 2.09 (0.87 to 5.06). Precautions taken to prevent contagion were not significantly associated with outcome, while, as expected, SARS-CoV-2-positive patients more often reported close contacts with COVID-19 cases. Relevantly, arthritis treatment significantly impacted on the probability of SARS-CoV-2 infection. The use of GC indeed increased risk (OR [95% CI] 2.89 [1.26 to 6.62]), with a significant effect limited only to daily prednisone doses ≥ 2.5 mg. Similarly, the use of csDMARDs was associated with a trend towards higher odds of COVID-19, while the use of b/tsDMARDs tended to reduce risk. No clear relationships between HCQ and confirmed COVID-19 cases were observed.

**Table 3:** Associations of confirmed SARS-CoV-2 infection. Univariable analysis

	OR	95% CI	***p***
Age ≥ 58 years	1.12	0.49 to 2.55	0.79
Male gender	1.04	0.44 to 2.46	0.93
Smoking	1.36	0.45 to 4.12	0.59
BMI			
Underweight	Reference		
Normal weight	0.46	0.05 to 3.88	0.48
Overweight	0.46	0.05 to 4.18	0.49
Obese	0.79	0.08 to 7.69	0.84
Hypertension	2.09	0.87 to 5.06	0.10
Diabetes	1.28	0.29 to 5.58	0.74
Home lockdown	0.62	0.26 to 1.50	0.29
Use of masks and gloves	0.64	0.27 to 1.51	0.30
Contact avoidance	0.73	0.31 to 1.75	0.48
Contacts with COVID-19	13.01	5.14 to 32.91	< 0.001
Diagnosis			
SpA	Reference		
PsA	0.37	0.07 to 2.03	0.25
RA	1.03	0.34 to 3.08	0.96
Disease duration ≥ 120 months	0.65	0.27 to 1.53	0.32
PDN	2.89	1.26 to 6.62	0.01
PDN dose			
0 mg/day	Reference		
< 2.5 mg/day	1.41	0.39 to 5.15	0.61
≥ 2.5 mg/day	4.22	1.74 to 10.23	0.001
HCQ	1.21	0.45 to 3.29	0.70
csDMARDs	1.80	0.76 to 4.27	0.18
b/tsDMARDs	0.46	0.20 to 1.06	0.07

*BMI* body mass index, *SpA* spondyloarthritis, *PsA* psoriatic arthritis, *RA* rheumatoid arthritis, *PDN* prednisone, *HCQ* hydroxychloroquine, *csDMARDs* conventional synthetic disease-modifying anti-rheumatic drugs, *b/ts DMARDs* biological/targeted synthetic disease-modifying anti-rheumatic drugs

In multiple bivariable models in which steroids, csDMARDs, and b/tsDMARDs were entered separately (Table 4), the use of GC was confirmed to independently predict increased risk of SARS-CoV-2 infection irrespective of comorbidities, precautions taken to prevent contagion, and contacts with COVID-19 cases. In contrast, the trends for higher odds of infection in csDMARD-treated and lower odds in b/tsDMARD-treated patients were apparently not independent from other variables. GC maintained independent association also in bivariable models including the other classes of anti-rheumatic drugs.

**Table 4:** Associations of confirmed SARS-CoV-2 infection. Bivariable analysis

	OR	95% CI	***p***	AUC
**Hypertension**				
Hypertension	1.73	1.14 to 2.64	0.01	0.66
PDN	3.04	2.09 to 4.43	< 0.001	
Hypertension	2.04	1.18 to 3.53	0.01	0.61
csDMARDs	1.74	0.53 to 5.72	0.37	
Hypertension	1.81	1.47 to 2.24	< 0.001	0.64
b/tsDMARDs	0.46	0.18 to 1.21	0.12	
**Home lockdown**				
Home lockdown	0.55	0.35 to 0.85	0.008	0.67
PDN	3.20	1.97 to 5.18	< 0.001	
Home lockdown	0.62	0.40 to 0.95	0.03	0.59
csDMARDs	1.58	0.66 to 3.77	0.31	
Home lockdown	0.63	0.46 to 0.85	0.002	0.63
b/tsDMARDs	0.51	0.11 to 2.29	0.38	
**Contacts with COVID-19**				
Contacts with COVID-19	12.74	2.38 to 68.30	0.003	0.74
PDN	3.14	1.72 to 5.73	< 0.001	
Contacts with COVID-19	13.33	2.08 to 85.40	0.006	0.68
csDMARDs	1.77	0.60 to 5.21	0.30	
Contacts with COVID-19	11.76	1.74 to 79.51	0.01	0.68
b/tsDMARDs	0.48	0.12 to 1.92	0.30	
**Treatment**				
PDN	2.70	1.34 to 5.46	0.006	0.65
csDMARDs	1.56	0.45 to 5.35	0.48	
PDN	2.67	1.84 to 3.86	< 0.001	0.67
b/tsDMARDs	0.51	0.18 to 1.44	0.21	
csDMARDs	1.72	0.51 to 5.84	0.38	0.62
b/tsDMARDs	0.47	0.15 to 1.53	0.21	

*PDN* prednisone, *csDMARDs* conventional synthetic disease-modifying anti-rheumatic drugs, *b/ts DMARDs* biological/targeted synthetic disease-modifying anti-rheumatic drugs

#### Highly suspicious infection

When the analysis was extended to include also highly suspicious SARS-CoV-2 infection, none of the demographic variables appeared consistently associated with the outcome. Again, the risk of SARS-CoV-2 infection appeared differently conditioned by the different classes of anti-rheumatic drugs. Patients on treatment with b/tsDMARDs were indeed significantly less affected, while the use of GC especially at doses ≥ 2.5 mg/day tended to increase the risk of infection (supplementary Table S3). No effects were seen in association with csDMARDs.

In multivariable analyses, treatment variables were confirmed as independent predictors of SARS-CoV-2 infection. In particular, the use of GC emerged as a risk factor also in this setting including milder cases, while b/tsDMARD-treated patients less frequently developed symptoms of COVID-19. Even when forced in the model, csDMARDs did not modify the odds of infection (Table 5).

**Table 5:** Associations of confirmed or highly suspicious SARS-CoV-2 infection. Multivariable analysis

	OR	95% CI	***p***
Disease duration ≥ 120 months	0.71	0.50 to 0.99	0.04
Home lockdown	1.02	0.64 to 1.60	0.94
PDN	1.23	1.04 to 1.44	0.02
csDMARDs	0.89	0.55 to 1.43	0.62
b/tsDMARDs	0.47	0.46 to 0.48	< 0.001
**AUC 0.62**			

*PDN* prednisone, *csDMARDs* conventional synthetic disease-modifying anti-rheumatic drugs, *b/ts DMARDs* biological/targeted synthetic disease-modifying anti-rheumatic drugs. *Highly suspicious infection:* respiratory symptoms + contacts with COVID-19 cases

#### Unlikely infection

We then tested the associations of patients’ and disease characteristics with a more permissive definition of SARS-CoV-2 infection also including patients with acute respiratory symptoms in the absence of known contacts with COVID-19 cases. As shown in Supplementary Table S4, no significant associated factors emerged among demographic and clinical characteristics apart from higher odds in younger patients. In contrast to confirmed and highly suspicious SARS-CoV-2 infection, none of the classes of anti-rheumatic drugs conferred increased or reduced risk. The neutral impact of GC and b/tsDMARDs was confirmed even when treatments were forced in multivariable analyses. Relevantly, after exclusion of confirmed and highly suspicious COVID-19 cases, the use of b/tsDMARDs was instead associated with a trend for increased odds of respiratory symptoms after adjustment for age and GC co-medication (OR [95% CI] 1.26 [0.94 to 1.69], *p* = 0.12).

## Discussion

This study first investigated the impact of chronic GC therapy and other immunosuppressive drugs on the prevalence of COVID-19 in a large cohort of patients with immune-mediated inflammatory arthritis and answered some crucial questions about the management of patients with IMIDs during the pandemic.

The first message that clearly emerges from our analysis is the close correlation between the development of SARS-CoV-2 infection and chronic GC treatment, especially at dose > 2.5 mg daily. Although short-term randomized controlled trials (RCTs) exploring the impact of low-dose GC have documented little or no increase in infectious risk [42], evidences from several observational studies confirm the facilitating effect of GC on the occurrence of various infections. Most of these studies have stratified the study population in relation to the GC dose (low, medium, and high) and concluded for an increased risk of both common and severe infections, especially in patients receiving medium to high GC doses (more than 7.5 mg daily of prednisone equivalent) [43]. The largest and most recent observational study conducted on 275,072 adults prescribed GC orally within a primary care database (The Health Improvement Network) showed that the adjusted hazard ratios for infections with significantly higher risk in the GC-exposed population ranged from 2.01 (95% CI 1.83–2.19; *p* < 0.001) for cutaneous cellulitis to 5.84 (95% CI 5.61–6.08, *p* < 0.001) for lower respiratory tract infections [44]. The results of our analysis, in line with what already reported for other types of infection, confirm this trend also for COVID-19. Preliminary data from the Global Rheumatology Alliance physician-reported registry recently highlighted increased risk of hospitalization for COVID-19 among patients with a wide range of rheumatic diseases, including connective tissue diseases and vasculitis, receiving prednisone doses ≥ 10 mg/day [41]. Compared with systemic autoimmune diseases, patients with chronic inflammatory arthritis, like those of our analysis, are less often managed with GC and usually receive lower doses. The low number of hospitalizations seen in our study is therefore not unexpected. However, our data confirm the potential harmful effects of GC across the spectrum of SARS-CoV-2 infection. Indeed, even low doses of prednisone were strongly associated with increased rates of confirmed SARS-CoV-2 cases in our cohort and also conferred a slightly increased risk of milder infections who could not get access to swab-based confirmation at the peak of the pandemic. Given the high proportion of subjects chronically taking GC for IMIDs, the relevance of this information can certainly be crucial for the management of these fragile patients during the outbreak. However, our results should not encourage indiscriminate suspension of GC. Indeed, the outcomes of symptomatic SARS-CoV-2 infection were overall favorable in our cohort, underscoring the importance of a balanced benefit-risk assessment in every patient. Equally important, results from our and previous analyses do not contrast with the possible therapeutic role of GC in more advanced stages of COVID-19 dominated by hyper-inflammation [45, 46].

On the other hand, the use of powerful immunosuppressants such as ts/bDMARDs has been considered a potential additional risk for COVID-19 since the beginning of the outbreak [19, 47]. Based on the multitude of data available from RCTs [48, 49] and especially observational studies [50–52], the pro-infective role of this class of drugs compared with csDMARDs has been well established, with only small variations according to the different mechanism of action [23, 53]. Conversely, our data from this point of view are unexpectedly very reassuring to the point of revealing a potentially protective effect of ts/bDMARDs on the occurrence of COVID-19 in chronically treated patients. The improvement in knowledge on COVID-19-related ARDS pathogenesis has pointed out the central role of the abnormal immune response to SARS-CoV-2 leading to a massive release of pro-inflammatory mediators known as cytokine release storm [54, 55]. This has actually paved the way for the use of biological drugs and small molecules in the treatment of the most severe subsets of COVID-19 [13, 56–61]. Although the apparent beneficial therapeutic effects of immunomodulatory agents cannot be translated into any speculation on their prophylactic role, the possibility that chronic use of ts/bDMARDs may dampen exaggerated immune reactions, thus mitigating COVID-19 progression into more symptomatic patterns, deserves further investigation. For the same reason, the course of COVID-19 in our patients receiving ts/bDMARDs was mild overall, with less than 50% of patients requiring low-flow oxygen therapy, no hospitalization in intensive care units, and no deaths. Intriguingly, the use of ts/bDMARDs was associated with a trend for the occurrence of respiratory symptoms in the absence of known contacts with COVID-19 cases, a condition which we defined unlikely COVID-19. A number of other viral and bacterial respiratory pathogens were indeed circulating in our geographical area at the time of the current survey. This finding would therefore advance the fascinating hypothesis of a potential protective role of immunomodulating agents in SARS-CoV-2 infection, unlike other viral infections. In the absence of mechanistic data, however, the most plausible explanation for the reduced odds of symptomatic infection in ts/bDMARD-treated patients remains at present a stricter adherence to the measures to prevent contagion. Indeed, the effect of ts/bDMARDs on swab-diagnosed COVID-19 in our cohort was not independent of home lockdown. Further supporting a more cautious behavior, patients judging themselves more immunosuppressed declared stricter adherence to general measures even in case of home lockdown (use of masks and gloves 74.9% vs 70.2%, *p* = 0.14; contact avoidance 80.3% vs 69.7%, *p* < 0.001).

The relationship between COVID-19 and csDMARDs therapy is still unclear and controversial. In particular, the role of antimalarial drugs as a possible prophylactic therapy against SARS-CoV-2 infection has been hypothesized on the basis of the results of in vitro studies [62, 63], even though to date in vivo confirmation is still incomplete and controversial [38, 64–66]. The message we provided by our analysis did not confirm an effect of HCQ in the prevention of COVID-19. A potential confounder may lie in the HCQ regimen, which in current COVID-19 management protocols and RCTs provides for a loading dose of 600/800 mg per day in the early stages [67], which is not commonly used in the treatment of inflammatory arthritis and is very far from the dose used in our cohort (200/400 mg daily) [68]. In addition, our cohort included many patients being treated with other potentially active drugs on the risk of COVID-19 such as GC and ts/bDMARDs and may therefore not be optimal to test the actual role of antimalarial drugs in this area. Only studies conducted on populations free of confounding factors and randomized to receive or not HCQ will be able to really address this issue [69]. In contrast, our data confirm the reassuring safety profile of csDMARDs, and in particular of MTX at rheumatological doses, on serious infections [70]. The use of csDMARDs indeed did not appear to significantly impact on symptomatic SARS-CoV-2 infection in our cohort when a restrictive definition of cases was used.

The current study certainly has some limitations. The first one is linked to defining COVID-19 as confirmed or highly suspect entirely on the basis of what the patients stated in the survey. Although this aspect is a potential methodological weakness in the design of the study, the particular emergency situation in which the data collection was carried out did not allow the entire cohort to undergo a confirmatory analysis by swab or serological test. As a consequence, the study could not evaluate subjects with asymptomatic COVID-19 as they were not intercepted by our survey. The second one, intrinsic to a cross-sectional survey partially administered by telephone, is the possibility of having missed all those patients who could not respond to the survey because they were hospitalized or died due to the infection. However, the rate of non-responders we recorded was negligible (1.96%) and unlikely to significantly affect the overall results. It is meaningful that this very high response rate to the survey may have been facilitated by the lockdown imposed by health authorities in Lombardy since 9 March 2020, as it was easier to contact patients confined at home. Among these few non-responders, almost all patients who did not respond directly were confirmed alive and without symptoms of infection by a relative who answered our phone call. These patients were not included in the final study population but were surely not contributing to a significant distortion of the COVID-19 prevalence in our cohort. At the time of the survey, the only accepted used tool for a definite diagnosis was the nasopharyngeal swab, but only that part of the population affected by more severe or life-threatening subsets could be actually swabbed, leaving major doubts about the real prevalence of the infection. For this reason, we decided to broaden the definition to include subjects who had not had access to the swab but who presented symptoms consistent with COVID-19 having been in close contact with a confirmed COVID-19 case in the last 14 days prior to onset of symptoms, according to WHO criteria. This permitted the regression analysis to be conducted on a larger sample size, roughly confirming the same results observed on COVID-19 positive subjects only. After the close of our survey, serologic tests have become available. However, many unknowns persist regarding SARS-CoV-2 immunity and assay interpretation, including the extent of antibody responses in mild infections and in younger subjects [71]. In the absence of solid data on the performance and interpretation of serologic tests, the WHO definition of highly suspicious infection used here and in similar studies remains therefore valid.

## Conclusions

In conclusion, this study provides all specialists facing the COVID-19 emergency with a very reassuring message about the possibility of suggesting RMDs patients to continue their current therapy with ts/bDMARDs without an increased risk and probably with a milder infection course. Conversely, the use of GC especially a dose > 2.5 mg per day should be cautiously evaluated during the pandemic.

## Supplementary Information

**Additional file 1.**

## Data Availability

Study protocol, statistical analysis, and individual participant data that underlie the results reported in this article, after deidentification (text, tables, figures, and appendices) will be available beginning 6 months and ending 5 years following article publication for investigators whose proposed use of the data has been approved by an independent review committee.

## Abbreviations

ARDS: Acute respiratory distress syndrome
ASDAS: Ankylosing Spondylitis Disease Activity Score
AUC: Area under the receiver operating characteristic curve
bDMARDs: Biologic disease-modifying anti-rheumatic drugs
COVID-19: Coronavirus disease 19
csDMARDs: Conventional-synthetic disease-modifying anti-rheumatic drugs
DAPSA: Disease Activity in Psoriatic Arthritis
DAS28: Disease Activity Score 28
GC: Glucocorticoids
HCQ: Hydroxychloroquine
JAK: Janus kinases
IMIDs: Immune-mediated inflammatory diseases
IQR: Interquartile range
LFN: Leflunomide
MTX: Methotrexate
OR: Odds ratio
PsA: Psoriatic arthritis
RA: Rheumatoid arthritis
RCTs: Randomized controlled trials
RMDs: Rheumatic and musculoskeletal diseases
SARS-CoV-2: Severe acute respiratory syndrome-coronavirus-2
SD: Standard deviation
SpA: Spondyloarthritis
SSZ: Sulfasalazine
TNF: Tumor necrosis factor
tsDMARDs: Targeted-synthetic disease-modifying anti-rheumatic drugs
UA: Undifferentiated arthritis
WHO: World Health Organization
95% CI: 95% confidence interval
