# Supplementary material for: Impact of corticosteroids and immunosuppressive therapies on symptomatic SARS-CoV-2 infection in a large cohort of patients with chronic inflammatory arthritis
Source: Arthritis Res Ther. 2020 Dec 30;22:290. doi: 10.1186/s13075-020-02395-6 (PMC7772957; doi:10.1186/s13075-020-02395-6)
Supplement: Supplementary file 1 — Additional file 1. [file 13075_2020_2395_MOESM1_ESM.docx]

**Supplementary Table S1. Treatment disposition of the study population**

|  | **Total**  **n=2.050** | **RA**  **n=1.228** | **UA**  **n=127** | **PsA**  **n=398** | **SpA**  **n=297** |
| --- | --- | --- | --- | --- | --- |
| b/tsDMARDs cohort, n. (%) | 1.278 (62.3) | 735 (59.9) | 0 (0) | 291 (73.1) | 252 (84.8) |
| monotherapy, n. (%) | 588 (46) | 247 (33.6) | - | 148 (50.9) | 193 (76.6) |
| csDMARDs, n. (%)  MTX, n. (%)  SSZ, n. (%)  LFN, n. (%)  CYA, n. (%)  others, n. (%) | 625 (48.9)  519 (83)  59 (9.4)  40 (6.4)  13 (2.1)  3 (0.5) | 428 (58.2)  367 (85.7)  18 (4.2)  36 (8.4)  8 (1.9)  2 (0.5) | - | 140 (35.2)  113 (80.7)  24 (17.1)  3 (2.1)  5 (3.6)  0 (0) | 57 (19.2)  38 (66.7)  17 (29.8)  1 (1.8)  0 (0)  1 (1.8) |
| HCQ, n. (%) | 133 (10.4) | 123 (16.7) | - | 8 (2) | 2 (0.7) |
| non b/tsDMARD cohort, n. (%) | 772 (37.7) | 493 (40.1) | 127 (100) | 107 (26.9) | 45 (15.2) |
| neither csDMARDs nor HCQ | 178 (23.1) | 89 (18.1) | 38 (29.9) | 24 (22.4) | 27 (60) |
| csDMARDs  MTX, n. (%)  SSZ, n. (%)  LFN, n. (%)  CYA, n. (%)  others, n. (%) | 423 (54.8)  370 (87.5)  39 (9.2)  13 (3.1)  10 (2.4)  1 (0.2) | 305 (61.9)  282 (92.5)  14 (4.6)  9 (3)  5 (1.6)  1 (0.3) | 24 (18.9)  17 (70.8)  7 (29.2)  0 (0)  0 (0)  0 (0) | 77 (72)  61 (79.2)  11 (14.3)  3 (3.9)  4 (5.2)  0 (0) | 17 (37.8)  10 (58.8)  7 (41.2)  1 (5.9)  1 (5.9)  (0) |
| HCQ | 249 (32.3) | 161 (32.7) | 72 (56.7) | 13 (12.1) | 3 (6.7) |

RA = rheumatoid arthritis; UA = undifferentiated arthritis; PsA = psoriatic arthritis; SpA = spondyloarthritis; b/tsDMARDs= biological/targeted synthetic disease modifying anti-rheumatic drugs; csDMARDs = conventional synthetic disease modifying anti-rheumatic drugs; MTX = methotrexate; SSZ = sulfasalazine; LFN = leflunomide; CYA = cyclosporin A; HCQ = hydroxychloroquine.

**Supplementary Table S2. Characteristics of the study population by Centre**

|  | Total | | RA | | PsA | | SpA | |
| --- | --- | --- | --- | --- | --- | --- | --- | --- |
|  | Pavia  n=1.120 | Milan  n=930 | Pavia  n=664 | Milan  n=564 | Pavia  n=203 | Milan  n=195 | Pavia  n=126 | Milan  n=171 |
| Age, mean (SD), yrs | 59.9 (15.1) | 55.3 (14.3) | 63.7 (14.2) | 58.6 (14.4) | 56.5 (12.6) | 52.6 (12.3) | 47.6 (13) | 47.5 (12.5) |
| Female gender, n. (%) | 753 (67.2) | 601 (64.6) | 498 (75) | 445 (78.9) | 104 (51.2) | 92 (47.2) | 60 (47.6) | 64 (37.4) |
| Current smokers, n. (%) | 163 (15.9) | 113 (17.4) | 84 (13.7) | 63 (16) | 34 (18.8) | 23 (17.7) | 22 (19.6) | 27 (21.6) |
| BMI, mean (SD)  overweight, n. (%)  obese, n. (%) | 25.7 (5.1)  317 (33.9)  161 (17.2) | 24.5 (5.1)  208 (30.5)  72 (10.6) | 25.4 (5.2)  181 (32.4)  83 (14.8) | 24.1 (5)  123 (29.2)  47 (11.2) | 27 (5.2)  57 (36.5)  39 (25) | 24.9 (6.5)  45 (33.8)  19 (14.3) | 25.6 (4.5)  40 (42.1)  14 (14.7) | 24.2 (3.6)  40 (31.5)  6 (4.7) |
| Hypertension, n. (%) | 438 (41.8) | 205 (22) | 285 (45.7) | 145 (25.7) | 77 (41.8) | 36 (18.5) | 31 (26.7) | 24 (14) |
| Diabetes, n. (%) | 107 (10.2) | 51 (5.5) | 74 (12) | 35 (6.2) | 20 (10.9) | 11 (5.6) | 7 (6) | 5 (2.9) |
| Disease duration, median (IQR), mo | 96 (51-168) | 132 (72-240) | 120 (60-180) | 173 (72-240) | 108 (60-168) | 120 (60-216) | 108 (63-177) | 120 (60-225) |
| Use of PDN, n. (%) | 344 (30.8) | 297 (31.9) | 261 (39.5) | 250 (44.3) | 43 (21.2) | 27 (13.8) | 11 (8.7) | 20 (11.7) |
| PDN dose, mean (SD), mg/day | 4 (2.9) | 4.7 (3.7) | 3.9 (2.4) | 4.7 (3.6) | 4.9 (5.1) | 5 (2.2) | 4.3 (1.2) | 4.9 (5.6) |
| Use of HCQ, n. (%) | 281 (25.1) | 101 (10.9) | 192 (29) | 92 (16.3) | 14 (6.9) | 7 (3.6) | 3 (2.4) | 2 (1.2) |
| Use of csDMARDs, n. (%) | 627 (56.1) | 421 (45.3) | 433 (65.3) | 300 (53.2) | 131 (64.5) | 86 (44.1) | 39 (31) | 35 (20.5) |
| Use of b/tsDMARDs, n. (%) | 542 (48.4) | 736 (79.1) | 315 (47.4) | 420 (74.5) | 130 (64) | 161 (82.6) | 97 (77) | 155 (90.6) |

RA = rheumatoid arthritis; PsA = psoriatic arthritis; SpA = spondyloarthritis; BMI = body mass index; PDN = prednisone; HCQ = hydroxychloroquine; csDMARDs = conventional synthetic disease modifying anti-rheumatic drugs; b/tsDMARDs= biological/targeted synthetic disease modifying anti-rheumatic drugs.

Patients with undifferentiated arthritis (UA) only recruited from Pavia.

**Supplementary Table S3. Associations of confirmed or highly suspicious* SARS-CoV-2 infection. Univariable analysis**

|  | **OR** | **95% CI** | **p** |
| --- | --- | --- | --- |
| Age ≥58 yrs | 0.81 | 0.46 to 1.41 | 0.45 |
| Male gender | 0.86 | 0.47 to 1.56 | 0.62 |
| Smoking | 0.93 | 0.41 to 2.11 | 0.87 |
| BMI  underweight  normal weight  overweight  obese | reference  1.72  1.28  1.87 | 0.23 to 12.97  0.16 to 10.10  0.23 to 15.47 | 0.60  0.81  0.56 |
| Hypertension | 1.56 | 0.87 to 2.80 | 0.14 |
| Diabetes | 0.78 | 0.24 to 2.54 | 0.68 |
| Home lock down | 0.97 | 0.54 to 1.73 | 0.92 |
| Use of masks and gloves | 0.83 | 0.46 to 1.50 | 0.55 |
| Contact avoidance | 0.85 | 0.47 to 1.56 | 0.61 |
| Diagnosis  SpA  PsA  UA  RA | reference  0.49  1.04  0.88 | 0.17 to 1.39  0.31 to 3.44  0.42 to 1.87 | 0.18  0.95  0.75 |
| Disease duration ≥120 mo | 0.48 | 0.26 to 0.89 | 0.02 |
| PDN | 1.25 | 0.79 to 1.98 | 0.34 |
| PDN dose  0 mg/d  <2.5 mg/d  ≥2.5 mg/d | reference  0.97  1.51 | 0.50 to 1.88  0.88 to 2.58 | 0.93  0.13 |
| HCQ | 1.04 | 0.52 to 2.09 | 0.91 |
| csDMARDs | 1.12 | 0.64 to 1.94 | 0.70 |
| b/tsDMARDs | 0.40 | 0.23 to 0.70 | 0.001 |

* fever and/or cough and/or dyspnea of recent onset in a patient having been in close contact with a confirmed COVID-19 case in the last 14 days prior to onset of symptoms

BMI = body mass index; SpA= spondyloarthritis; PsA = psoriatic arthritis; UA = undifferentiated arthritis; RA = rheumatoid arthritis; PDN = prednisone; HCQ = hydroxychloroquine; csDMARDs = conventional synthetic disease modifying anti-rheumatic drugs; b/ts DMARDs = biological/targeted synthetic disease modifying anti-rheumatic drugs.

**Supplementary Table S4. Associations of confirmed, highly suspicious or unlikely SARS-CoV-2 infection. Univariable analysis**

|  | **OR** | **95% CI** | **p** |
| --- | --- | --- | --- |
| Age ≥58 yrs | 0.57 | 0.45 to 0.74 | <0.001 |
| Male gender | 0.88 | 0.68 to 1.14 | 0.35 |
| Smoking | 1.12 | 0.78 to 1.59 | 0.54 |
| BMI  underweight  normal weight  overweight  obese | reference  0.87  0.64  0.92 | 0.44 to 1.72  0.32 to 1.29  0.44 to 1.92 | 0.69  0.22  0.82 |
| Hypertension | 1.00 | 0.77 to 1.31 | 0.98 |
| Diabetes | 1.08 | 0.69 to 1.69 | 0.74 |
| Home lock down | 0.95 | 0.74 to 1.21 | 0.66 |
| Use of masks and gloves | 0.88 | 0.69 to 1.13 | 0.32 |
| Contact avoidance | 1.03 | 0.80 to 1.34 | 0.80 |
| Diagnosis  SpA  PsA  UA  RA | reference  0.72  0.56  0.74 | 0.48 to 1.07  0.31 to 1.04  0.53 to 1.02 | 0.11  0.07  0.07 |
| Disease duration ≥120 mo | 0.96 | 0.75 to 1.22 | 0.72 |
| PDN | 0.90 | 0.69 to 1.17 | 0.42 |
| PDN dose  0 mg/d  <2.5 mg/d  ≥2.5 mg/d | reference  0.73  1.06 | 0.50 to 1.06  0.77 to 1.45 | 0.10  0.74 |
| HCQ | 0.97 | 0.71 to 1.32 | 0.83 |
| csDMARDs | 1.18 | 0.93 to 1.50 | 0.18 |
| b/tsDMARDs | 1.16 | 0.90 to 1.49 | 0.26 |

BMI = body mass index; SpA= spondyloarthritis; PsA = psoriatic arthritis; UA = undifferentiated arthritis; RA = rheumatoid arthritis; PDN = prednisone; HCQ = hydroxychloroquine; csDMARDs = conventional synthetic disease modifying anti-rheumatic drugs; b/ts DMARDs = biological/targeted synthetic disease modifying anti-rheumatic drugs.
